# Association of COVID-19 mortality with politics and on-demand testing in 217 U.S. counties

**DOI:** 10.1186/s12889-021-12063-2

**Published:** 2021-12-02

**Authors:** Leon S. Robertson

**Affiliations:** Yale University School of Public Health, New Haven, CT USA

**Keywords:** COVID testing, COVID politics, COVID mortality, COVID risk factors

## Abstract

**Background:**

Previous research found increased COVID-19 spread associated with politics and on-demand testing but not in the same study. The objective of this study is to estimate the contribution of each corrected for the other and a variety of known risk factors.

**Methods:**

Using data from 217 U.S. counties of more than 50,000 population where testing data were available in April, 2021, the associations of COVID-19 deaths with politics, testing and other risk factors were examined by Poisson and least squares regression.

**Results:**

Statistical controls for 15 risk factors failed to eliminate the association of COVID mortality risk with percent of vote for Donald Trump in 2016 or negative tests per population. Each is independently predictive of increased mortality.

**Conclusion:**

Apparently, many people who test negative for the SARS-CoV-2 virus engage in activities that increase their risk, a problem likely to increase with the availability of home tests. There is no association of negative tests with the Trump vote but, according to polling data, Trump voters’ past resistance to public health recommendations has been extended to resistance to being vaccinated, threatening the goal of herd immunity.

## Background

COVID-19, the disease caused by a new strain of coronavirus (SARS-CoV-2) first detected in China in 2019, began to spread rapidly in the U.S. in March 2020. By mid-April, more than 2000 people per day in the U.S. died from complications of the disease. Research on cumulative COVID-19 mortality in the U.S. at the end of May, 2020 found that deaths attributed to the disease were highly concentrated in densely populated counties predictable by indicators of crowding and other social and economic factors [1]. Forty-two percent of U.S. counties had no deaths attributed to COVID-19 by the end of May, 2020. Nevertheless, school closures, limits to size of gatherings, shelter-in-place orders, mask use, physical distancing, and other countermeasures were usually required throughout states that adopted mandates, resulting in relatively more resentment of these mandates in less populated areas [2].

The issue of when and where to require countermeasures became politically partisan early on. The Republican President, Donald J. Trump, made numerous statements in White House briefings and on social media that contradicted scientific evidence and supported protesters who advocated against countermeasures [3]. Internet hackers based in Russia spread misinformation about the pandemic on social media websites, attempted to steal information about vaccine development, and disabled computers in hospitals treating COVID-19 patients, demanding ransom to restore them [4].

The cities where the virus was initially most prevalent were largely governed by mayors who were members of the Democratic Party. Mayors and governors in a given state often disagreed on the necessity and extent of deployment of countermeasures, especially so if they were from different political parties. In late Spring and early Summer of 2020 many countermeasures were abandoned and the infections and deaths surged in a wider swath of counties.

A Republican governor and a greater percentage of Trump voters was more predictive of adoption of or timing of physical distancing mandates than number of cases in a state at the time [5]. As early as March, 2020, self-reported behavior to protect from the virus was related to partisan views. Democrats were more likely to claim compliance with recommended practices [6]. During April–June, 2020, self-reported resistance to physical distancing declined among Democrats but increased among Republicans through mid-June before declining slightly [7]. In another study, mask use among U.S. counties was found inverse to percent of the 2016 vote for Trump [8].

As more tests for infection by the virus became available, testing sites were inaugurated where people were allowed to be tested whether or not they had symptoms or suspected they had been exposed. Once it was established that infected but asymptomatic people could spread the virus, those who advocated such testing thought that mass testing was necessary to identify the infected and trace their contacts. If the infected would self-quarantine for 2 weeks, they would not spread the virus. Many of the testing sites could not keep up with demand. There were not enough personnel to do the tracing. A study of contact tracing in 13 U.S. health departments in 11 states and an Indian Health Service unit found that less than 60% of people who tested positive were interviewed and only a third named contacts. Of the contacts who were traced, less than half agreed to follow up [9]. Public health agencies suffered from lack of funding for years leading up to the inadequate response to the pandemic [10].

A time-series study of testing in various countries and U.S. states found that hospitalizations for COVID-19 increased 2 weeks after negative tests rose in jurisdictions where on- demand testing was allowed. That finding lent support to the hypothesis that people who tested negative were engaging in activities that increased their exposure to such an extent that, whatever the benefit of tracing the contacts of those who tested positive, the benefit was more than offset. In countries that tested only people in high-risk groups, lower hospitalizations were found 2 weeks after increases in tests [11].

Testing advocates cited New Zealand and Slovakia as examples of the effectiveness of testing [12]. New Zealand did not allow on-demand testing. Slovakia changed from testing the symptomatic and vulnerable to on-demand testing in October, 2020 with disastrous results. Slovakia’s COVID-19 deaths per population were among the lowest of national rates during March–October, 2020; then soared to be among the highest during November, 2020 through April, 2021 [13].

The purpose of this paper is to report research based on two hypotheses. Adjusted for other risk factors: 1. Cumulative COVID-19 mortality in U.S. counties as of mid-April, 2021 was higher in relation to the percent of voters who voted for Donald Trump in 2016. 2. Cumulative COVID-19 mortality was higher in relation to greater numbers of negative tests.

## Data and methods

Data on cumulative tests and number of positive cases and deaths are available for 217 counties with 50,000 or more population in the U.S. states of California, Florida, New York, Pennsylvania, Texas, and Washington [14]. The number of negative tests in each county was obtained by subtracting total cases from total tests. To control statistically for other factors predictive of COVID-19 mortality, data on 15 factors that were found predictive in previous research as well as the percent who voted for Donald Trump in 2016 [15] were matched to the testing and mortality data from the 217 counties.

The data included five factors that increase the probability of human interaction that would facilitate spread of a contagious virus transmitted by human breath: population density per square kilometer, average number of persons per household, average employees per business, average religious adherents per congregation, and average number of social acquaintances per person reported in a population survey. Four factors that are known to be related to the severity of the disease were included separately: percent of the population with obesity, diabetes, elderly cardiovascular hospitalizations, and persons 65 years and older. Social and economic factors that are often related to health status were also included: percent of adults with at least a high school education, median age of the population, percent unemployment, median family income, income inequality, and percent African American or Hispanic ethnicity.

Population per square mile was downloaded from the U.S. Census Bureau [16] and converted to square kilometers. Estimated 2019 population, percent unemployed, and median household income prior to the pandemic for each county were downloaded from the U.S. Department of Agriculture website based on estimates from the U.S. Census Bureau and Bureau of Labor statistics [17]. Persons per household, social acquaintances, high school graduates, economic inequality, percent 65 years or older, percent with diabetes, and percent obese were downloaded from files accumulated from various sources by the Robert Wood Johnson Foundation [18]. Medicare hospital discharges for cardiovascular diseases were obtained from CDC Wonder [19]. Numbers of religious adherents and congregations were obtained from the Association of Religious Data Archives [20]. Numbers of businesses and employees were downloaded from the Bureau of Labor Statistics [21]. Percent African American and Hispanic were obtained from Dr. Randel Olson’s website [22].

The associations of politics and testing to COVID-19 mortality, corrected statistically for the influence of the other risk factors, were estimated by Poisson regression. In addition, a dummy variable was introduced for 5 of the 6 states to adjust for the aggregate effect of differences in mitigation efforts among the states. Logarithms or square roots, as appropriate, were performed on variables that had skewed frequency distributions. Log (population) was included as an offset variable to correct for differences in population size among the counties. The form of the regression equation is:

Accumulated number of COVID-19 deaths as of April 20, 2021 = b1 (Percent of the vote for Donald Trump in 2016) +.

b2 ((Negative tests for SARS-CoV-2)/1,000,000).

b3 (log (estimated 2019 residents per square kilometer)) +.

b4 (average number of persons per household) +.

b5 (log (average employees per business enterprise))+.

B6 (log (average religious adherents per congregation))+.

B7 (log (average number of social acquaintances.

reported per person)) +.

b8 (percent of the population that is obese) +.

b9 (percent of the population with diabetes) +.

b10 (Medicare cardiovascular hospitalization discharges.

2015–2017) +.

b11 (log (percent of the population 65 years or older)) +.

b12 (percent of adults who finished high school +.

b13 (log (median family income before the pandemic)) +.

b14 (income inequality before the pandemic) +.

b15 (percent unemployed before the pandemic) +.

b16 (√Percent African American) +.

b17 (√Percent Hispanic) +.

b18 (1 if California, else 0) +.

b19 (1 if Florida, else 0) +.

b20 (1 if New York, else 0) +.

b21 (1 if Pennsylvania, else 0) +.

b22 (1 if Texas, else 0).

The other state, Washington, was not included because the model would be over specified. Coefficients on the states are adjustments relative to Washington state, corrected for the other risk factors. When several of the risk factors were found intercorrelated, the association of the risk factors with percent Trump vote and negative tests per population were analyzed using ordinary least squares regression to assess their potential effect on conclusions regarding the hypotheses.

## Results

The results of the Poisson regression are presented in Table 1. COVID-19 deaths were higher in counties with more negative SARS CoV-2 tests and a higher percent of the 2016 vote for Donald Trump. Each of the indicators of increased crowding were predictive of more deaths. Of the three indicators of preexistent conditions known to increase severity of the disease, increases in deaths were associated with a greater percentage of persons with diabetes but the opposite was true of percent obese in a county. The Medicare cardiovascular discharge rate was not statistically significant. More deaths were associated with a larger percent of the population 65 years or older, a greater percent of African Americans and Hispanics and a lower percent of the population with a high school or better education. The economic indicators predicted more deaths in counties with lower median family income, greater income inequality and lower percent unemployment prior to the pandemic.

**Table 1:** Poisson Regression Coefficients and 95 Percent Confidence Intervals of Hypothesized Predictors of COVID-19 Mortality Among 217 U.S. Counties, April, 2021

	COVID-19 Deaths (95% C.I.)
Negative tests	.030 (.024, .036)
Percent vote Trump	.010 (.009, .011)
Log (population/square kilometer)	.020 (.011, .029)
Average persons per household	1.786 (1.729, 1.843)
Log (Average employees per business)	.011 (−.006, .026)
Log (Average religious per number of congregations)	.231 (.206, .256)
Log (claimed social acquaintances)	.031 (.006, .056)
Percent obese in the population	−.009 (−.012, −.006)
Percent diabetic in the population	.050 (.043, .057)
Cardiovascular hospital discharge rate	−.001 (−.002, .001)
Median age	.027 (.023 .031)
Percent aged 65 and older	.028 (.025, .031)
Percent adults finished high school	−.006 (−.008, −.004)
Log (median family income)	−1.065(− 1.017, − 1.113)
Income inequality	.046 (.033, .059)
Percent unemployed before COVID-19	−.360 (−.399, −.321)
Percent African American	.025 (.018, .032)
Percent Hispanic	.035 (.025, .045)
California	−.114 (−.150, −.078)
Florida	.037 (−.011, .085)
New York	.412 (.374, .450)
Pennsylvania	.479 (.434, .524)
Texas	−.046 (−.092, −.003)
Intercept	−3.68

Since each factor is measured on a different scale from the others, the magnitude of the regression coefficients does not represent relative importance.

Since regression coefficients can be distorted by correlation among predictor variables, the regression estimates of negative SARS CoV-2 tests and percent Trump vote were examined in relation each to each other as well as the other predictor variables. The results are in Table 2. Negative tests per population and percent Trump vote were not significantly correlated. Negative tests were weakly correlated to percent obesity (inverse), percent of adults who finished high school, median family income and income inequality, but not strongly enough to be concerned about the coefficient on negative tests. These weak associations could not distort the coefficient substantially. A relatively higher percent Trump vote was correlated to lower population density per square kilometer, fewer occupants per residence, more diabetics per population, more cardiovascular hospitalizations, higher percent of high school graduates, lower income inequality and lower percent Hispanics in the population. These correlations are strong enough to raise concern regarding the coefficient on the Trump vote.

**Table 2:** Least Squares Regression of Negative SARS COV-2 Tests Per Million Population and Percent Trump Vote by Hypothesized Predictors of COVID-19 Mortality, 217 U.S. Counties, April 2021

	Log (Negative Tests/million population) (95% C.I.)	Percent Trump Vote (95% CI)
Percent Trump vote	−.006 (−.016, .004)	
Log (population/square kilometer)	−.120 (−.012, .252)	− 3.89 (− 5.560,− 2.220)
Average persons per household	−.345 (− 1.207, .489)	− 14.298 (− 29.496,− 3.100)
Log (Average employees per business)	0.050 (−.200, .304)	1.099 (− 2.306, 4.504)
Log (Average religious per number of congregations)	.337 (−.078, .752)	3.864 (− 1.639, 9.367)
Log (claimed social acquaintances)	−.103 (−.473, .266)	2.011 (− 2.900, 6.920)
Percent obese in the population	.044 (.005, .083)	.137 (−.378, .652)
Percent diabetic in the population	−.015 (−.116, .086)	1.726 (.405, 3.047)
Cardiovascular hospital discharge rate	−.010 (−.023, .003)	.031 (.136, .484)
Median age	−.029 (−.088, .030)	−.461 (− 1.246, .324)
Percent aged 65 and older	.002 (−.043, .039)	.030 (−.520, .580)
Percent adults finished high school	−.028 (−.045, −.010)	.340 (.109, .571)
Log (median family income)	.985 (.269, 1.701)	2.929 (− 6.951, 12.449)
Income inequality	.320 (.119, .521)	− 5.550 (− 8.094, − 3.006)
Percent unemployed before COVID-19	−.249 (−.740, .242)	− 7.614 (− 14.060, − 1.168)
Percent African American	.021 (−.097, .039)	− 0.333 (− 1.904, 1.239)
Percent Hispanic	−.070 (−.256, −.117)	− 2.845 (− 5.285, −-.403)
Intercept	−9.048	46.704
R^2^	.23	.68

## Discussion

The results further support the hypothesis that on-demand testing resulted in behavior that increased spread of the SARS-CoV-2 virus by those whose tests were negative. Apparently, many ignored warnings that a negative test did not justify travel or other behavior that increased risk of exposure. The statistical control for numerous other risk factors failed to erase the association of COVID-19 deaths with testing.

Even without the adverse behavioral effect, on-demand testing is clearly not necessary to contain spread of the virus. For example, as of April, 2021, New Zealand had a COVID-19 death rate of 5 per million population compared to 1742 per million population in the U.S. [23]. New Zealand tested only those with symptoms and was much more efficient than the U.S. in enforcing quarantines of incoming travelers and orders to shelter in place, physically distance and wear masks [24]. New Zealand is more urbanized than the U.S.; 87% of the population lives in urban areas compared to 82% in the U.S. [25].

In the U.S., home test kits became available in a variety of retail outlets in April, 2021. The Centers for Disease Control and Prevention recommends that they be used only by those who think they may have been exposed to the virus and that the results of the test should be reported to their health care practitioner [26]. Given the results of on-demand testing thus far, many users are unlikely to follow those recommendations. More convenient availability of the tests is likely to lead to increased risky behavior of those who test negative. SARS CoV-2 RNA can become part of the human genome such that people who have been infected test positive long after they are no longer a risk to others [27]. To the extent that is the case, required testing to return to work, school or engage in other activities will become discriminatory. Control of the virus in the U.S. and most countries is now substantially dependent on the extent of vaccinations and the prospect that vaccines will be effective against mutating variants as well as continued physical distancing and mask use in higher risk situations.

## Conclusions

The pace of vaccinations in the U.S. slowed in April, 2021 partly due to resistance apparently based on politics. In a March, 2021 CBS News poll more than a third of Republicans said that they would refuse to be vaccinated compared to 30% of independents and 10% of Democrats [28].

The results of the present study suggest that politically based resistance to COVID-19 countermeasures contributed to the spread of the virus. Although the covariation of percent Trump votes with several other risk factors raises doubts about the precise extent of politics as a factor relative to other factors, the noted polls and studies as well as videos of Trump political rallies and White House events during the pandemic indicate Trump supporters’ more frequent failure to take precautions. The correlations of the trump vote with COVID-19 risk factors indicate that Trump voters disproportionately reside in counties that were at lower risk. Had they complied with preventive recommendations, they likely would have remained so. The lack of correlation between Trump’s 2016 vote and testing among counties suggests that his supporters did not include testing as a symbol of resistance. If they and others continue to resist masks, physical distancing and vaccinations, the goal of herd immunity is jeopardized.

The major limitation of this study is reliance on correlations among aggregated data in counties. Inference of individual behavior from aggregated data occasionally leads to inaccurate conclusions about behavior of individuals in the aggregated groups [21].

## Data Availability

The datasets used and/or analyzed during the current study are available from the corresponding author on reasonable request.
